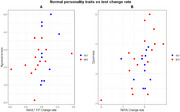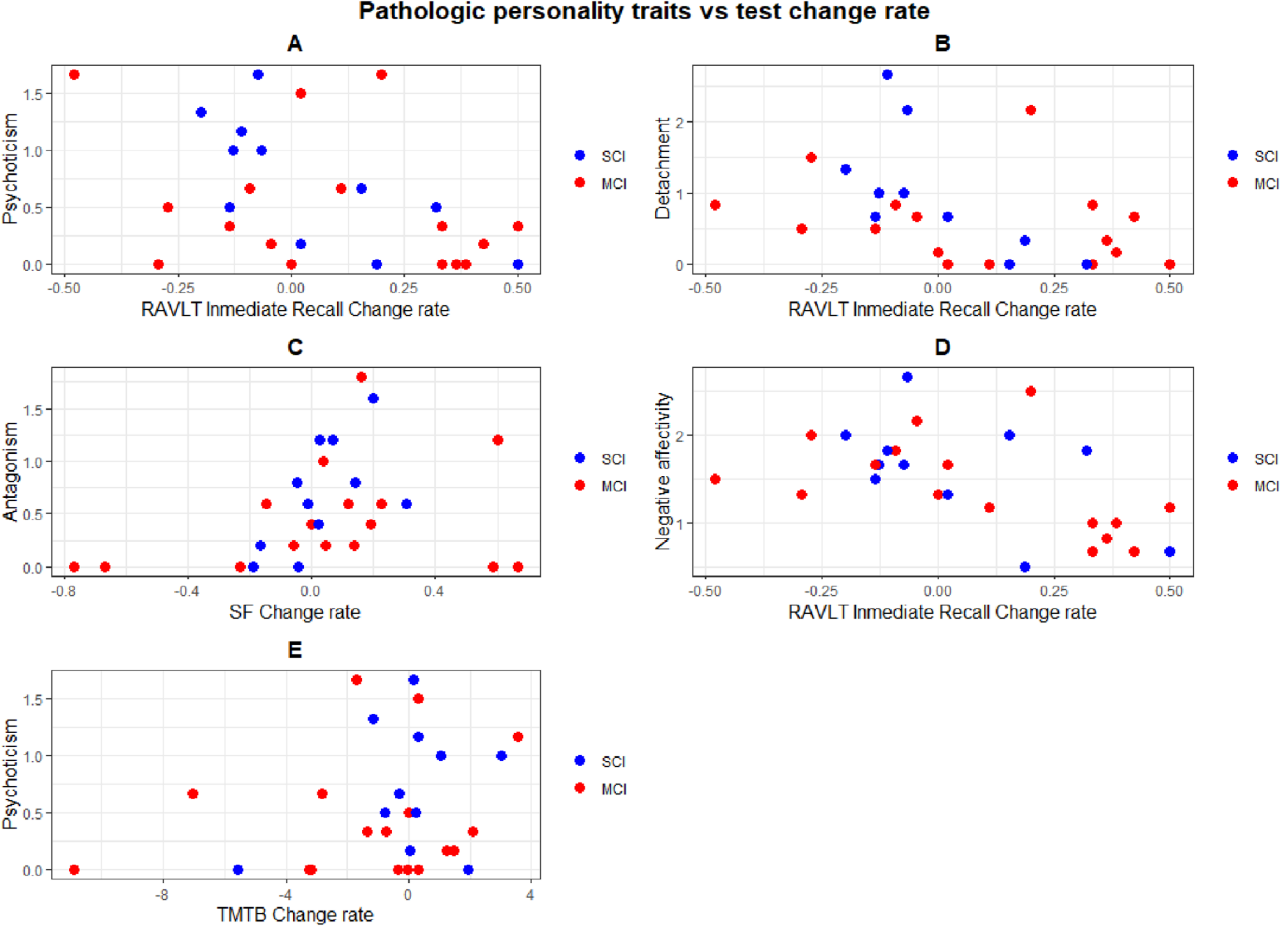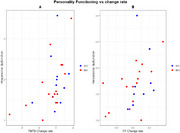# Exploring the link between personality traits and cognitive change in older adults with mild and subjective cognitive impairment

**DOI:** 10.1002/alz70858_099415

**Published:** 2025-12-25

**Authors:** Enio Alejandro Yabur Carranza, Camila Morgenstern, Macarena Alvarez Cortina, Natalia Sierra Sanjurjo, Santiago O'Neill

**Affiliations:** ^1^ Neuroscience Institute Favaloro Foundation, Buenos Aires, Argentina; ^2^ Neuroscience Institute, Favaloro Foundation, Buenos Aires, Argentina

## Abstract

**Background:**

The interplay between personality, particularly pathological traits, and cognitive change in older adults with Subjective Cognitive Impairment (SCI) and Mild Cognitive Impairment (MCI) remains unclear. This study aims to describe the relationship between normal and pathological personality and cognitive change in older adults with SCI and MCI.

**Method:**

A correlational longitudinal study was conducted with 29 older adults (11 SCI, 18 MCI) from the Favaloro Foundation Neuroscience Institute. Participants completed three self‐report personality questionnaires: the Big Five Inventory, the Personality Functioning Scale and the Personality Inventory for DSM‐5 Brief Form Plus. Patients with psychiatric disorders, dementia, or epilepsy were excluded. Participants underwent two comprehensive neuropsychological assessments over at least two years. A change index was calculated for each test. Spearman correlations were determined between the personality traits and the cognitive change index using Rstudio 4.4.1.

**Result:**

Participants were 6 men and 23 women with a mean age of 72.7 years. Agreeableness correlated positively with the change rate in false positives on a memory recognition task (*p* = 0.04, rho=0.39). Psychoticism, negative affectivity and detachment were negatively correlated with the change index in immediate recall (*p* = 0.0441, rho=‐0.47; *p* >0.01, rho=‐0.53; *p* >0.01, rho=‐0.51, respectively). Openness correlated positively with the change index on a processing speed test (*p* > 0.01, rho = 0.5). Detachment and intrapersonal dysfunction correlated positively with the change index on an alternation task (*p* = 0.02, rho=‐0.43; *p* >0.01, rho=0.35). Antagonism correlated positively with semantic fluency (*p* = 0.03, rho=0.36), and interpersonal dysfunction with phonological fluency (*p* = 0.017, rho=0.46).

**Conclusion:**

To our knowledge, this is the first study to quantify the relationship between normal and pathological personality traits and cognitive change in SCI and MCI using a comprehensive neuropsychological assessment. These findings suggest that normal and pathological personality traits might influence cognitive performance over time, particularly on tasks related to memory and attention. Understanding this influence can inform clinical interventions and contribute to personalized care for these individuals.